# Myxedema Ascites: An Unusual Presentation of Uncontrolled Hypothyroidism

**DOI:** 10.7759/cureus.2627

**Published:** 2018-05-14

**Authors:** Rohit Dhingra, Puja Rai, Jakob Sieker, Jatin Roper

**Affiliations:** 1 Department of Medicine, Tufts Medical Center, Boston, USA; 2 Department of Pathology, Tufts Medical Center, Boston, USA; 3 Gastroenterology, Tufts Medical Center, Boston, USA

**Keywords:** hypothyroidism, ascites, myxedema, liver

## Abstract

We describe a case of myxedema ascites in a 64-year-old male with a history of hypothyroidism noncompliant with medical therapy who presented with syncope, hematemesis, melena, and abdominal distension. The patient received intravenous levothyroxine with a good response and improved upon discharge. This case highlights the importance of considering hypothyroidism as an etiology of unexplained ascites. The analysis of ascites from myxedema may not always have a significantly elevated protein (>2.5g/dL). Appropriate diagnosis should also rely on the clinical presentation along with a rapid and positive response to thyroid hormone replacement therapy.

## Introduction

Primary hypothyroidism is a very common diagnosis, affecting 3.8%-4.6% of the general population [1]. Patients classically present with symptoms such as weight gain, constipation, and fatigue. However, up to 4% of patients with significantly uncontrolled hypothyroidism may develop abdominal ascites [2]. Since the differential diagnosis for ascites is broad, myxedema ascites should be considered in a patient with a history of hypothyroidism and ascites. The diagnosis is confirmed with a clinical response to thyroid hormone replacement, leading to a complete resolution of the ascites. An ascites fluid analysis typically demonstrates elevated protein (>2.5 g/dL) and low white blood cell (WBC) counts, mainly consisting of lymphocytes [2].

## Case presentation

**Figure 1:**
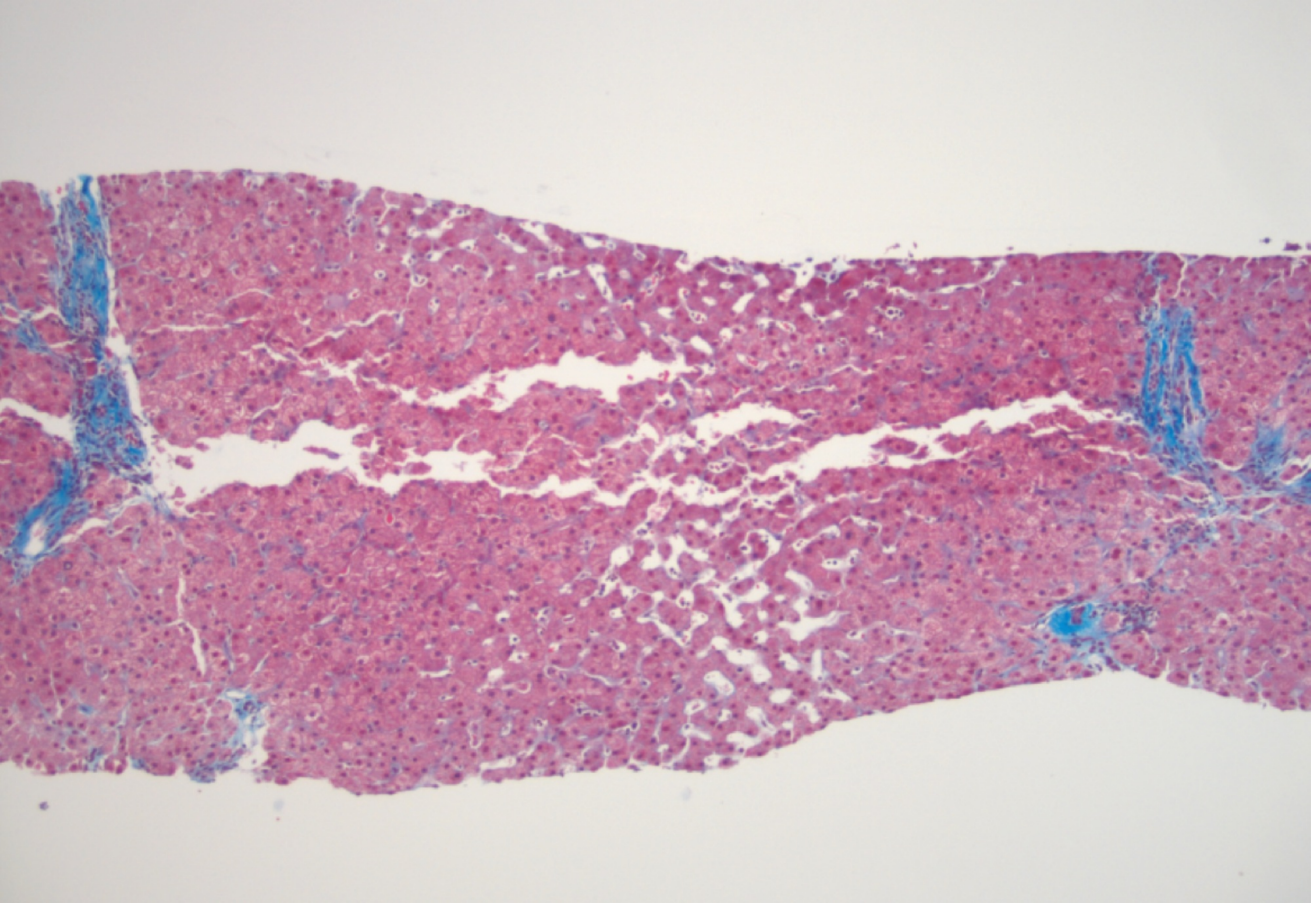
Liver Core Biopsy Liver biopsy with dilated sinuses, liver plate atrophy, and sinusoidal fibrosis, suggestive of chronic venous outflow obstruction

A 64-year-old male, with a past medical history of gastroesophageal reflux disease, alcohol abuse, and hypothyroidism non-compliant with medications, presented after a syncopal episode and several days of hematemesis, melena, and abdominal distension. The patient began to develop multiple daily episodes of vomiting of black liquid and melena four days prior to presentation, with associated lightheadedness and shortness of breath. He reported that he had previously been taking omeprazole, but stopped taking all medications several months prior. He reported taking ibuprofen for the past few weeks, consuming 400-1200 mg per day for one to two weeks for chronic back pain. The physical examination was notable for a significantly distended abdomen with findings consistent with ascites, which was reportedly new for him. Laboratory testing showed low hemoglobin (Hgb: 6.4 g/dL, which worsened to 5.1 g/dL over the same day), elevated aspartate aminotransferase (AST: 104 IU/L), significantly elevated thyroid stimulating hormone level (TSH: 60 units/mL), and an undetectable free thyroxine level (FT4). He was given two units of blood and was started on intravenous (IV) levothyroxine and hydrocortisone. He was admitted to the intensive care unit (ICU) and underwent an upper endoscopy, which showed an adherent clot in the distal esophagus, just proximal to a hiatal hernia in the distal esophagus, and received three clips and an epinephrine injection. A right upper quadrant ultrasound was performed, which demonstrated clear yellow fluid and fluid analysis notable for nucleated cells: 150/uL, neutrophils: 0%, mesothelial cells: 4%, lymph: 57%, monocytes: 38%, protein: 2.0 g/dL, albumin: 1.2 g/dL, lactate dehydrogenase (LDH): 106 IU/L, serum albumin: 3.1 g/dL, and serum-ascites albumin gradient (SAAG): 1.9. Infectious workup was also performed on the ascites, which was overall negative. However, amylase and lipase were not checked on the fluid. He then underwent a transjugular liver biopsy and hepatic venous pressure gradient measurement, which showed a normal liver, without any signs of cirrhosis (liver fibrosis 1/6) (Figure 1), and a normal hepatic venous pressure gradient (HVPG) of 3 mm Hg. The diagnosis of myxedema ascites secondary to longstanding hypothyroidism was presumed based on the history of untreated hypothyroidism and lack of portal hypertension or cirrhosis. During his hospitalization, the ascites clinically improved over eight days after the initiation of 300 mcg of IV levothyroxine and titrated to 150 mcg PO levothyroxine daily, which he continued upon discharge.

## Discussion

In this patient, the history of alcohol abuse, ascites, gastrointestinal bleeding, and SAAG>1.1 were suggestive of portal hypertension secondary to alcoholic cirrhosis. However, the normal HVPG ruled out portal hypertension as a cause of ascites, and the normal liver biopsy ruled out cirrhosis. The differential diagnosis for ascites without portal hypertension includes peritoneal malignancies and infections including tuberculosis, hypoalbuminemia, pancreatic disease, systemic lupus erythematosus, ovarian disease, and myxedema. Therefore, an empiric diagnosis of myxedema ascites was made.

Primary hypothyroidism presenting as ascites is rare and should be considered in any patient with a history of hypothyroidism. The mechanism responsible for the development of ascites in patients with hypothyroidism remains unclear but has been linked to increased capillary permeability and the loss of plasma proteins, causing a decrease in oncotic pressure and a buildup of fluid to occur in various body cavities. One study suggested that patients with hypothyroidism have low levels of nitric oxide and high levels of vascular endothelial growth factor (VEGF), which can also synergistically increase the permeability of capillaries and, thus, further allow plasma proteins to be lost from the intravascular space [3].

There have been several well-documented cases of myxedema ascites. In one case, a 71-year-old male presented with increased abdominal girth and anorexia for about 10 days. Ascitic fluid was notable for elevated total protein (3.5 g/dL) and low SAAG (0.8 g/dL) with gram stain and cytology negative. A laparoscopic biopsy of the peritoneum and liver were performed, revealing intrahepatic cholestasis without liver cirrhosis and a nonspecific histology of the peritoneum. An echocardiogram was done to exclude cardiac disease, which was normal. To prevent further fluid retention, the patient was treated with diuretics, with poor response and persistent symptoms. Therefore, thyroid testing was performed and revealed hypothyroidism. Thyroid replacement therapy was initiated, with a resolution of his ascites [2]. A literature review of 51 reported cases suggests that myxedema ascites typically manifests as a high protein level (>2.5 g/dL), with a mean of 3.9 g/dL. The mean SAAG was 1.5 g/dL with a range of 0.8-2.3 g/dL and a predominance of lymphocytes [2]. A consistent feature in every case has been a good response to thyroid replacement therapy with complete regression of the ascites. However, as demonstrated in this case, a high total protein level is not a definitive feature of this disease, and ascites fluid results should be correlated with clinical suspicion.

As with any new presentation of ascites, a comprehensive diagnostic workup is warranted, including history and a physical examination to evaluate for heart failure, alcohol abuse, signs of portal hypertension, and ascites analysis. Our case demonstrates that a SAAG of greater than 1.1 is not always indicative of portal hypertension, contrary to most cases seen [4]. In our case, both HVPG and liver biopsy were required to exclude portal hypertension and chronic liver disease. Due to the presumed theory of increased loss of plasma proteins, patients with myxedema from hypothyroidism have usually been reported to have an elevated total protein in the ascites fluid (usually >2.5 g/dL), mildly elevated SAAG (1.5 mg/dl), and lymphocytic predominance (>80%) [3], as demonstrated in Table 1. However, the fluid analysis of our patient was unusual in that it resulted in a lower than expected protein of 2.0 g/dL and higher than expected SAAG of 1.9 g/dL with a liver biopsy that did not show cirrhosis, further suggesting that the ascites was primarily due to hypothyroidism and not portal hypertension. It is important to note that the liver biopsy does potentially indicate other possible etiologies for an elevated SAAG, including pre-sinusoidal hypertension. For patients in which myxedema ascites is suspected, rapid clinical response to thyroid hormone replacement therapy will confirm the diagnosis.

**Table 1:** Summary of Patients with Myxedema Ascites Table demonstrating that most patients with myxedema ascites cited in case reports were found to have ascites with either protein >2.5 g/dL and/or mean serum-ascites albumin gradient (SAAG) 1.5 g/dL, distinct from our case with ascites demonstrating lower than expected protein (2.0 g/dL) and higher than expected SAAG (1.9 g/dL)

Patient #	Ascites Protein (g/dL)	SAAG (g/dL)	Reference #
1	3.5	0.9	[5]
2	4.1	1	[6]
3	3.5	0.8	[2]
4	2.4	1.7	[7]
5	2.1	0.8	[8]
Our Case	2.0	1.9	

## Conclusions

Myxedema ascites should be considered in patients with hypothyroidism and ascites, even in the setting of elevated SAAG and low protein in an ascites analysis. Conversely, ascites in patients with a normal liver biopsy and normal HVPG should prompt an evaluation of thyroid hormone levels.
